# Does the Hyperthermal Sarcomeric Oscillations Manifested by Body Temperature Support the Periodic Ventricular Dilation With Each Heartbeat?

**DOI:** 10.3389/fphys.2022.846206

**Published:** 2022-03-28

**Authors:** Seine A. Shintani

**Affiliations:** Department of Biomedical Sciences, College of Life and Health Sciences, Chubu University, Kasugai, Japan

**Keywords:** hyperthermal sarcomeric oscillations, contraction rhythm homeostasis, robustness of heartbeat, cardiac physiology, sarcomere

**FIGURE 1:**
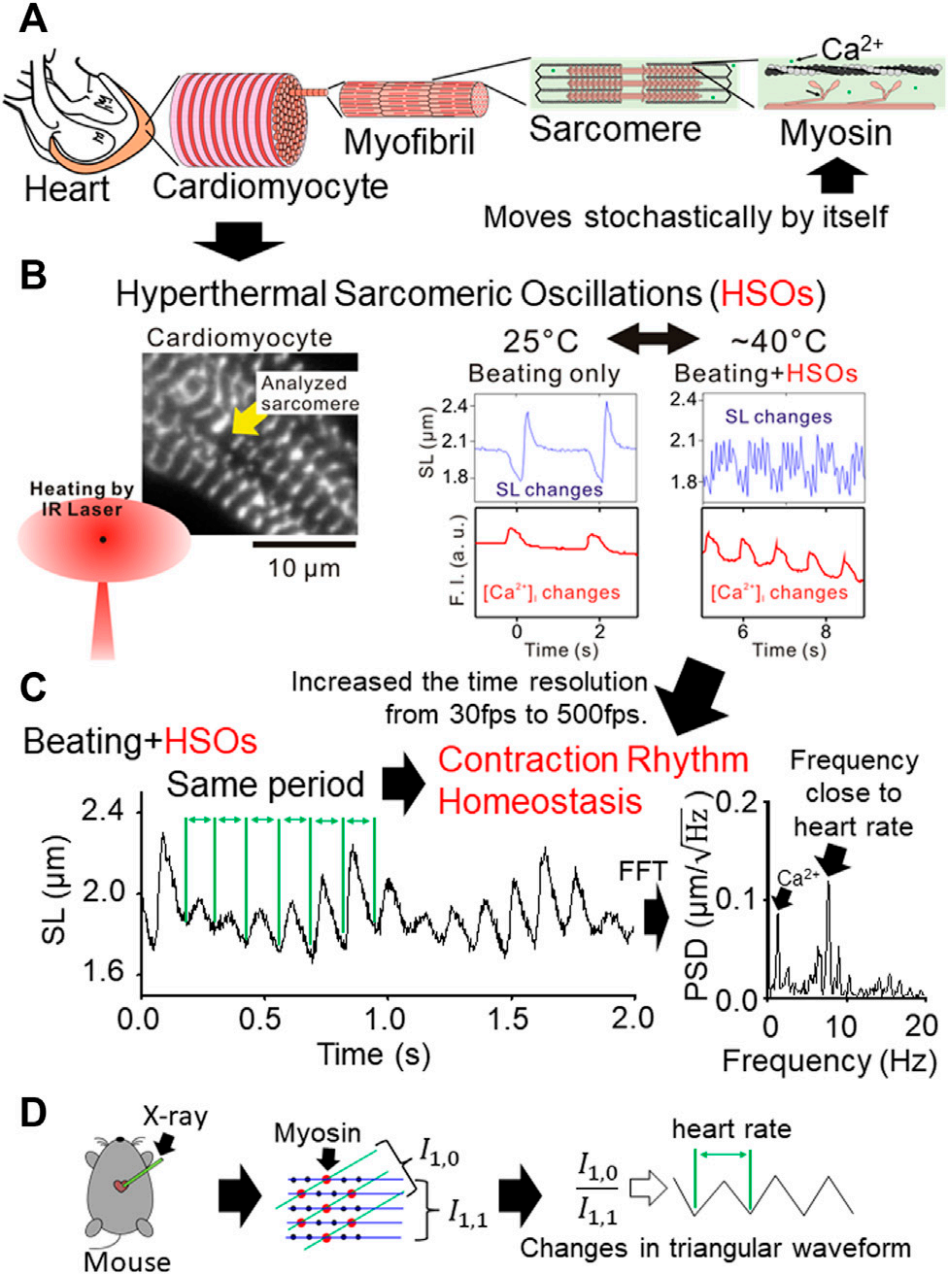
**(A)** Schematic diagram of the hierarchical structure from the myosin molecule, which can move only stochastically by itself, to the heart, which has a strong heartbeat. **(B)** Schematic diagram of the features of HSOs revealed in 2015 (Shintani et al., 2015). Fluorescence microscope image of cardiomyocytes that expresses α-actinin-AcGFP fusion protein in Z-line and enables measurement of sarcomere length (left panel). The cardiomyocytes were reversibly warmed by irradiating the vicinity of the cardiomyocytes with infrared rays having a wavelength that water absorbs well (left panel). Time-series changes in sarcomere length and intracellular calcium concentration before and after heating (right panel). By warming, HSOs with a higher frequency than the change in calcium concentration were discovered (right panel). **(C)** Schematic diagram of the characteristics of HSOs revealed in 2020 (Shintani et al., 2020). When the time resolution of sarcomere length measurement was improved from 30 fps to 500 fps, it became clear that the period of a sarcomeric oscillation cycle was kept constant even though the waveform of the sarcomeric oscillation changed significantly with the change in calcium concentration (left panel). The frequency of HSOs was close to the heart rate (right panel). **(D)** Schematic diagram of a non-invasive real-time measurement of the movement of myocardial contractile protein molecules from the beating heart by synchrotron radiation X-ray diffraction (Pearson et al., 2007). When the sarcomere contracts, the $I_{0,1}/I_{1,1}$ value decreases, and when the sarcomere relaxes, the $I_{0,1}/I_{1,1}$ value increases.

The heart transforms the chemical-mechanical reaction of myosin molecules, which move only stochastically by itself, into a robust heartbeat rhythm by its hierarchical structure (Figure 1A, Ishiwata et al., 2017). Myocardial pulsation is thought to be regulated by changes in calcium concentration in cardiomyocytes triggered by electrical excitement from cardiac pacemaker cells (Eisner et al., 2017). However, on the other hand, it has also been found that the heartbeat frequency is too high to sufficiently reduce the calcium concentration in cardiomyocytes that has increased due to muscle contraction (Eisner et al., 2017). The myocardium is known to be contracted when the intracellular calcium concentration is high (Eisner et al., 2017). So why can the heart relax quickly in the early diastole of each heartbeat, even though the calcium concentration in cardiomyocytes is high and the left ventricular pressure is low? Since the left ventricular pressure is low, there is little force to pull and lengthen the sarcomere. And since the calcium concentration in cardiomyocytes is still high, sarcomere generates force. Despite this, the reason why rapid relaxation to fill the ventricle with blood throughout diastole is possible may be that sarcomere has the property of periodically and rapidly relaxing and dilating even if the calcium concentration is still high.

In recent years, we have discovered that sarcomere in cardiomyocytes warmed to body temperature becomes an oscillation state that repeats contraction and relaxation (Figure 1B, Shintani et al., 2015). We named the sarcomere oscillations manifested by the heat of this body temperature HSOs (Hyperthermal Sarcomeric Oscillations). We believe that HSOs are the key to answering the previous question. Experiments with isolated cardiomyocytes have shown that HSOs enable rapid lengthening of sarcomere even at high intracellular calcium concentrations, and that the HSOs cycle is close to the heartbeat cycle (Figure 1C, Shintani et al., 2020). Given the rapid lengthening seen in HSOs during diastole of the heart, it explains the rapid lengthening of the heart despite high cardiomyocyte calcium levels and low left ventricular pressure.

HSOs are observed at 37°C–43°C in cardiomyocytes where calcium concentration fluctuates at about 1 Hz (Shintani et al., 2015; Shintani et al., 2020). Inhibiting calcium concentration fluctuations narrowed the temperature at which HSOs can be observed to 38°C–43°C (Shintani et al., 2015). To put it very simply, this may be due to a decrease in base calcium concentration. Conversely, when calcium concentration fluctuations higher than 1 Hz, such as heartbeat, occur, the base calcium concentration increases (Eisner et al., 2017), and the minimum temperature required to induce HSOs is likely to be lower than 37°C. The core body temperature of the working heart is higher than other parts of the body. Since even a mere core body temperature is measured at 37–38°C (Yoda et al., 2000), it is highly possible that the HSOs characteristics of sarcomere are manifested at the core body temperature of the working heart, which is considered to be higher.

It has been reported that sarcomere becomes an oscillation state that repeats contraction and relaxation under a solution with intermediate conditions between contraction and relaxation (Fabiato and Fabiato, 1978; Okamura and Ishiwata, 1988; Linke et al., 1993; Sasaki et al., 2006; Shintani et al., 2014; Shintani, 2021). Details are given in the cited paper, but “intermediate conditions between contraction and relaxation” are solution conditions in which sarcomere can exert a contractile force of about half of the maximum contractile force. In previous studies of sarcomeric oscillations, sarcomere was observed in a solution with a fixed calcium concentration (Ishiwata et al., 2017). The discovery of HSOs revealed that by warming cardiomyocyte sarcomere, it responds to changes in calcium concentration and at the same time performs sarcomeric oscillations with a cycle different from that of changes in calcium concentration (Shintani et al., 2015; Shintani et al., 2020). Moreover, sarcomere in the HSOs state has a CRH (Contraction Rhythm Homeostasis) that keeps the period constant, although the amplitude and waveform of the sarcomeric oscillation change significantly with changes in calcium concentration (Shintani et al., 2020). We named CRH the property of the contractile rhythm of HSOs that keeps the period constant even though the waveform changes in response to changes in calcium concentration. As mentioned above, sarcomere in the HSOs state, which repeats contraction and relaxation in a cycle close to the heartbeat, has a calcium concentration change responsiveness (Shintani et al., 2015; Shintani et al., 2020). Therefore, it is thought to synchronize in response to changes in calcium concentration in the heart cycle derived from the periodic excitement of cardiac pacemaker cells. We suspect that this synchronization of calcium concentration changes and mechanical sarcomeric oscillations is the basic state of the myocardial contractile system that supports the heartbeat.

In fact, in a mathematical model that reproduces HSOs, both sarcomere elongation and cardiac diastolic elongation of HSOs were movements based on the chained reversal stroke of myosin within the sarcomere (Washio et al., 2017; Washio et al., 2019; Shintani et al., 2020; Yoneda et al., 2021). It was a mathematical model-predicted expectation that myosin’s chained reversal strokes would cause sarcomeric oscillations (Washio et al., 2017). However, after that, it was experimentally confirmed that the reverse swing motion of myosin II existed (Fujita et al., 2019; Hwang et al., 2021).

Synchrotron radiation X-ray diffraction is a method that can measure the movement of myocardial contractile protein molecules from the beating heart in real time non-invasively (Pearson et al., 2007). In the measurement results by this synchrotron radiation X-ray diffraction method, it has been confirmed that the degree to which the myosin head moves and binds to actin with the heartbeat changes in a triangular waveform (Figure 1D, Pearson et al., 2007). Previously known sarcomeric oscillations at constant calcium concentrations were saw waveforms with slow contraction and rapid extension, but HSOs can also be oscillated in triangular waveforms with similar contraction and extension (Figure 1C, Shintani et al., 2020). The degree to which the myosin head migrates and binds to actin correlates with changes in sarcomere length. Therefore, the measurement results of the synchrotron radiation X-ray diffraction method suggest that the sarcomere in the heart continuously contracts and relaxes in a triangular waveform. Since HSOs can also continuously contract and relax with a triangular waveform of the same cycle, it is highly possible that the heartbeat utilizes the HSOs characteristics (Figures 1C,D).

From the above experimental facts, we believe that the beating heart uses HSOs to enable rapid relaxation to fill the ventricle with blood throughout diastole with each beating. And I think that the synchronization of HSOs with CRH and calcium fluctuation supports the robustness of the heartbeat.
